# Larval antibiosis to cabbage stem flea beetle ( *Psylliodes chrysocephala* ) is absent within oilseed rape ( *Brassica napus* )

**DOI:** 10.1002/ps.70808

**Published:** 2026-04-14

**Authors:** Ryan E Brock, Clara Courtney, Steven Penfield, Rachel Wells

**Affiliations:** ^1^ Department of Crop Genetics John Innes Centre Norfolk UK; ^2^ Institute of Organismic and Molecular Evolution Johannes Gutenberg University Mainz Germany

**Keywords:** antibiosis, Brassicaceae, cabbage stem flea beetle, entomology, host plant resistance, integrated pest management, oilseed rape, plant–insect interactions

## Abstract

**BACKGROUND:**

Insect pests present a global threat to crops, with plant resistance representing a key breeding goal. The cabbage stem flea beetle (*Psylliodes chrysocephala*; CSFB) is a key pest of oilseed rape (*Brassica napus*; OSR) in Europe; however, CSFB resistance is yet to be found in *B. napus*. To address this, we examine CSFB larval development over time, explore antibiosis across a genetically diverse *B. napus* panel, and test whether larvae can develop in model Brassicaceae species (*Brassica rapa* and *Arabidopsis thaliana*).

**RESULTS:**

CSFB larvae completed development from 4 weeks post‐infestation, undergoing a 20‐fold size increase, with larval recovery after 2 weeks allowing semi‐high‐throughput resistance phenotyping. Applying this method to 98 Brassicaceae genotypes (97 *B. napus* and 1 *Sinapis alba*), we found weak evidence for genotype effects on larval survival. However, phenotype validation with ‘resistant’ and ‘susceptible’ *B. napus* genotypes showed no differences in larval survival or adult emergence. Larval antibiosis was consistently observed in *S. alba*. Finally, we showed that model *B. rapa* and *A. thaliana* genotypes represent suitable hosts for CSFB, with larvae increasing eight to ten times in size after 2 weeks.

**CONCLUSION:**

CSFB larval antibiosis appears absent in *B. napus*, possibly because of bottlenecks experienced during domestication. However, larval antibiosis is present in *S. alba*, and future work should study the basis of this resistance. Further, CSFB larval screening in Brassicaceae model species presents an opportunity to explore CSFB resistance genetics, informing breeding progress for insect resistance in *B. napus*. © 2026 The Author(s). *Pest Management Science* published by John Wiley & Sons Ltd on behalf of Society of Chemical Industry.

## Graphical abstract

We screened a panel of 98 Brassicaceae genotypes (97 *Brassica napus* and 1 *Sinapis alba*) for cabbage stem flea beetle (*Psylliodes chrysocephala*) larval antibiosis. We found limited evidence for antibiosis across *B. napus* but consistent antibiosis in *S. alba*. Brassicaceae relatives may therefore better inform resistance traits for breeding.

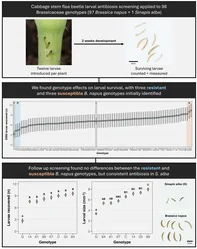

## 1 INTRODUCTION

Insect herbivory is one of the most common trophic interactions on earth and represents a major selection pressure for plant defence traits.^1^, ^2^, ^3^ In food crops, selective breeding for high yield, quality traits and uniform growth has reduced crop genetic diversity and altered plant–insect interactions,^4^, ^5^, ^6^ such that modern crops are generally more insect‐susceptible than their wild relatives.^7^, ^8^ In addition, agricultural ecosystems often comprise monocultures of crop genotypes, further increasing insect susceptibility.^9^ Insect pests therefore represent a global threat to food production, causing assumed yield losses of up to 40% a year.^10^, ^11^

Pesticide application broadly limits pest impacts on crop yield and enhances agricultural productivity^12^, ^13^ (although see Perrot *et al*.^14^). However, the adverse environmental effects of pesticide use^15^, ^16^ coupled with the evolution of pesticide‐resistant insect populations^17^ mean more sustainable pest management approaches are required. Host plant resistance, defined as plant traits influencing insect‐associated damage,^18^ offers a promising strategy for pest management.^19^, ^20^, ^21^ Plant resistance to insect herbivory can manifest through three non‐exclusive pathways: (i) antixenosis, in which insects are deterred from host plant colonisation; (ii) antibiosis, in which insects suffer reduced fitness on host plants; and (iii) tolerance, in which plants compensate for insect damage through increased growth or reproduction.^18^, ^21^, ^22^ A crucial first step in identifying host plant resistance requires phenotyping for resistance variation across plant species or genotypes, before using genetic mapping approaches to associate genes and traits with resistance.^20^, ^21^ However, the identification of insect resistance genes has proven challenging because of the polygenic nature of resistance and labour‐intensive phenotyping methodologies.^21^, ^23^, ^24^

Oilseed rape (*Brassica napus*; OSR), an allopolyploid derived from the hybridisation of *Brassica rapa* and *Brassica oleracea*,^25^ represents the second‐largest oilseed crop by area grown globally and is a key break crop in rotation systems.^26^, ^27^ An important breakthrough in OSR commercial success was the production of double‐low genotypes,^25^, ^28^, ^29^ plants yielding seeds with low levels of both erucic acid, a fatty acid with potential cardiotoxic effects,^30^ and glucosinolates, a group of secondary metabolites involved in plant defence.^31^ As a result, modern OSR genotypes are severely bottlenecked and express low levels of glucosinolates within tissues,^25^, ^32^, ^33^, ^34^, ^35^ compromising disease and pest resistance. OSR is therefore susceptible to a range of pathogens and insects,^36^, ^37^ with insect resistance now representing a major objective for breeders.^23^, ^38^

In Europe, the cabbage stem flea beetle (*Psylliodes chrysocephala*; CSFB), a Brassicaceae specialist,^39^ represents a key pest of OSR.^37^ The CSFB life cycle is closely linked to the cropping cycle of winter OSR, with distinct damage symptoms associated with different life stages.^40^ Adult CSFBs feed on emerging seedlings during early autumn, impacting crop establishment and reducing vigour.^40^ Following adult reproduction, CSFB larvae mine OSR stems and petioles during autumn and winter, passing through three larval instars before leaving the plant to pupate in spring.^40^ CSFB larvae are the more economically damaging life‐stage, with mining leading to stunted growth, frost and pathogen susceptibility, and plant mortality, causing severe yield penalties.^41^, ^42^, ^43^, ^44^

CSFB control in OSR is heavily pesticide reliant. Previously, management involved the use of neonicotinoid seed treatments that provided systemic protection throughout the growth cycle.^45^, ^46^ However, concerns over the environmental effects of neonicotinoids have led to usage restrictions in the European Union since 2013.^47^, ^48^ In response, growers have become increasingly reliant on pyrethroid insecticides,^45^, ^46^ driving the evolution of pyrethroid resistance in European CSFB populations.^49^, ^50^, ^51^ Limited control options have therefore led to increased CSFB abundance and subsequent reductions in OSR growth.^46^, ^52^, ^53^ In the UK, a tenfold increase in CSFB larval loads^52^ has been accompanied by a 67% reduction in OSR land use (from 621 000 ha in 2014 to 204 000 ha in 2025)^54^ following the neonicotinoid ban. Given policies aimed at reducing pesticide use,^55^ the development of CSFB‐resistant OSR genotypes represents a key target for the European OSR industry.^23^, ^38^

Reported attempts to phenotype for CSFB resistance in Brassicaceae have been extremely limited to date, with just a single study comparing larval resistance between *B. napus* and four relatives (*B. rapa*, *B. oleracea*, *Sinapis alba* and *Raphanus sativa*).^56^ This is mirrored for other insect pests of *B. napus* (reviewed in Hervé^23^ and Obermeier *et al*.^38^), reflecting the challenging nature of exploring insect resistance. Despite phenotyping relatively small Brassicaceae collections, these studies have highlighted a lack of insect resistance across *B. napus* genotypes, partial resistance in some diploid relatives (*B. rapa* and *B. oleracea*) and re‐synthesised *B. napus* genotypes, and resistance and/or tolerance in more distantly related Brassicaceae species.^23^, ^38^ CSFB larval antibiosis has been shown in *S. alba*, with larvae experiencing lower survival and slower development, although such effects were lacking across four tested *B. napus* genotypes.^56^ However, whether CSFB larval antibiosis exists across a more genetically diverse *B. napus* population remains to be seen.

In the current study, we aimed to explore CSFB larval antibiosis (defined as reduced survival and development) across a genetically diverse *B. napus* population, with the goal of identifying resistant genotypes that may provide trait and gene discovery for future breeding efforts. We outline a method for CSFB larval screening^57^ and investigate larval development under laboratory conditions, with our results informing a semi‐high‐throughput approach for phenotyping CSFB larval antibiosis across 98 Brassicaceae genotypes (97 *B. napus* and 1 *S. alba*). Importantly, this represents the largest *B. napus* collection phenotyped for insect resistance to date. Given the phenotypic diversity in this collection,^58^, ^59^, ^60^ we hypothesised that larval antibiosis would exist in some *B. napus* genotypes, providing a foundation for association mapping of genes involved in CSFB resistance. Finally, to broaden the genetic resources available for CSFB–Brassicaceae interactions, we applied CSFB larval screening to two model Brassicaceae species, *B. rapa* and *Arabidopsis thaliana*.

## 2 MATERIALS AND METHODS

### 2.1 Cabbage stem flea beetle rearing

We established laboratory CSFB populations by rearing larvae from OSR (KWS Barbados) collected at the John Innes Centre Field Station (Bawburgh, UK) in the winters of 2021/2022 and 2022/2023. Larvae were recovered from OSR plants using the Berlese method^61^ and placed on pak choi (*B. rapa chinensis* Hanakan; Suttons Seeds, Paignton, UK) to complete development. On emergence, adult beetles were kept in populations of up to 70 individuals inside ventilated boxes (length × width × height: 17 × 12 × 6 cm) lined with moist tissue and fed Chinese cabbage (*B. rapa pekinensis* Hilton; Suttons Seeds) *ad libitum*. Fresh cabbage was provided every 7–12 days, during which dead adults were removed and eggs were collected. Collected eggs were placed on pak choi to undergo development, with newly emerged adults used to setup new populations, thereby completing the CSFB life cycle under laboratory conditions. All rearing steps were carried out under constant conditions [22 ± 1 °C, ~60% relative humidity (RH), 16:8 h light/dark photoperiod]. Captive CSFB populations would have cycled through between approximately three and nine generations when the below experiments were carried out, with no further addition of field‐collected individuals since winter 2022/2023.

### 2.2 Larval development time course

To understand CSFB larval development under laboratory conditions, we conducted a time course experiment across four time points (1–4 weeks after infestation) in a single *B. napus* genotype (Apex‐93_5 × Ginyou_3 DH). Seeds were sown into Levington Advance F2 compost (ICL, Amsterdam, The Netherlands) and 10 days post‐sowing, 20 seedlings were individually re‐potted into 9‐cm pots containing JIC Cereal Mix (65% peat, 25% loam, 10% grit, 3 kg/m^3^ dolomitic limestone, 1.3 kg/m^3^ PG Mix, 3 kg/m^3^ osmocote exact), assigned to a time point and randomised (Supporting Information, Fig. S1). Plants were grown under constant conditions [18 ± 1 °C, ~60% RH, 10:14 h light/dark photoperiod] and watered regularly. The same growth procedure and conditions were used for all other experiments conducted.

Larval infestation began 4 weeks post‐sowing, when plants possessed five or six true leaves (mean = 5.3). Infestation was based on Döring and Ulber^56^ and Coston *et al*.,^43^ with twelve <24‐h‐old CSFB larvae added to each plant over a 4‐day period (three larvae per day),^57^ reflecting field‐realistic UK infestation levels expected to lower harvest yield.^43^, ^52^, ^61^ Adding larvae across 4 days allows sufficient larvae to hatch, is field‐relevant (whereby larvae enter plants across multiple months),^42^ and ensures plants are not overly stressed from multiple larvae entering concurrently.

To stimulate hatching, ~1000 CSFB eggs were placed at constant conditions [22 ± 1 °C, ~60% RH, 16:8 h light/dark] 1–4 days before infestation. Each infestation day, three <24‐h‐old larvae were transferred to the base of each plant (Fig. 1(A)) and observed for 10 s to ensure movement. Any larva that did not move was removed and replaced with a new larva. Each plant was sealed in a microperforated bag (length × width: 70 × 38 cm), ensuring that larvae could not move between plants. To account for circadian effects on plant defence compounds,^62^ larval infestation was carried out at the same time every day, with plants infested in a different order each day. On the final infestation day, plants were checked for successful larval penetration, defined as entry holes and mines visible on the stem or petioles (Fig. 1(A)) and/or visible larvae under the plant epidermis.

Five infested plants were harvested and weighed at each time point, with the leaves, petioles and stem dissected to recover larvae. The location and instar of all live larvae in each plant were recorded, with recovered larvae stored in 70% ethanol until imaging. Dead larvae found during dissection were also recorded. Twenty <24‐h‐old larvae were also collected for imaging. All collected larvae were photographed (Supporting Information, Fig. S2(A),(B)), and then ImageJ v.1.52a^63^ was used to threshold the image and measure larval size (in mm^2^) with the *Analyze Particles* function (Supporting Information, Fig. S2(C),(D)). Occasionally, larvae were damaged during dissection and unavailable for measurement. Of the 131 live larvae recovered during dissections, 126 were measured.

### 2.3 Screening a genetically diverse *B. napus* population for larval antibiosis

To explore variation in CSFB larval antibiosis in *B. napus*, we conducted larval screening across 96 genetically fixed genotypes, comprising spring, winter and semi‐winter OSR, swede and kale morphotypes, from the Renewable Industrial Products from Rapeseed^58^ panel (Supporting Information, Table S1). Genotypes were randomised across a temporal 12‐block alpha design [plots per block (*k*) = 24, replicates per genotype (*r*) = 3, blocks per replicate (*s*) = 4] and grown across two CER shelves, with a new block beginning every 1–9 weeks (Supporting Information, Table S2 and Fig. S3). Each block also contained a negative *B. napus* control (KWS Campus) and a positive *S. alba* control (Elsoms G1), with CSFB larval antibiosis having been previously demonstrated in *S. alba*.^56^ In a single block, two genotypes died before infestation and were therefore regrown in later blocks. Hence, each block contained 24–27 plants, giving a total of 3 replicates for all 96 *B. napus* genotypes and 12 replicates for both control genotypes.

Larval infestation^57^ was performed at 4 weeks post‐sowing, when plants possessed between one and nine true leaves (mean = 4.32). Our larval development experiment showed highest larval recovery 2 weeks post‐infestation, with larvae increasing eight‐fold in size (Fig. 1(B),(C)). Hence, 2 weeks post‐infestation, all plants were harvested and weighed, with larvae recovered using the Berlese method^61^ at 22 ± 1 °C for up to 17 days. Of the 2229 larvae recovered, 2201 were imaged and measured as before. Larval antibiosis was defined from both the number of larvae recovered (larval survival) and the size of recovered larvae (larval development).

### 2.4 Phenotype validation

We identified potential variation in CSFB larval antibiosis across *B. napus* genotypes (Fig. 2(A)). Therefore, to confirm larval antibiosis variation, we selected six genotypes (three ‘resistant’ (33, 61 and 67) and three ‘susceptible’ (14, 84 and 89)) for phenotype validation, along with the two control genotypes used previously. A total of nine replicates per genotype were phenotyped across three temporal blocks (i.e. three genotype replicates per block, giving 24 plants per block and 72 plants overall). Plants were grown in randomised blocks across two CER shelves, with a new block starting every 1–3 weeks (Supporting Information, Fig. S4). Larval infestation^57^ was performed 3 weeks post‐sowing, when plants possessed between two and six true leaves (mean = 3.88). Two weeks post‐infestation, plants were harvested, weighed and larvae were collected using the Berlese method,^61^ with all recovered larvae (*n* = 561) imaged and measured.

To link larval recovery to adult development, we also explored adult emergence across the selected genotypes. Patterns of larval recovery between the control genotypes were similar across the *B. napus* population screen and phenotype validation experiment (Supporting Information, Fig. S5). Therefore, to increase throughput, the adult emergence experiment only contained the six *B. napus* genotypes of interest. Ten replicates for each genotype were phenotyped across two temporal blocks (i.e. 5 genotype replicates per block, giving 30 plants per block and 60 plants overall). Plants were grown in randomised blocks across two CER shelves, with a new block starting each week (Supporting Information, Fig. S6). Larval infestation^57^ was carried out 4 weeks post‐sowing, when plants possessed between three and six true leaves (mean = 4.25), and larvae were left to develop until adult emergence.

Based on larval development results (Fig. 1(B)–(D)), we stopped watering plants at 6 weeks post‐infestation (because all larvae should have left the plants to pupate) and began checking for adult emergence every 2–7 days. Seven weeks post‐infestation (when adults were first observed; Fig. 3(D)), plant vegetation was disposed of, allowing easier collection of adults. At each check, collected adults were counted and removed using an insect aspirator, with the experiment terminated when no new adults had emerged for 2 weeks. Hence, this experiment provided two extra measures of larval antibiosis: total adult emergence (the number of larvae that successfully completed development) and time to adult emergence (larval development time). One replicate of genotype 61 died between infestation and adult collection and so was excluded from analyses.

A full protocol for CSFB larval antibiosis phenotyping can be found in Brock and Wells.^57^

### 2.5 Larval screening in Brassicaceae model species

*Brassica napus* relatives may offer novel insights into CSFB resistance by providing tractable genetic resources.^23^, ^56^ Hence, we assessed whether CSFB larval screening worked in reference *B. rapa* and *A. thaliana* genotypes.

*Brassica rapa* larval screening was carried out using the R‐o‐18 genotype, a rapid‐cycling yellow mustard (*B. rapa trilocularis*) with genetic resources that include a high‐quality genome and sequenced TILLING (targeting induced local lesions in genome) population.^64^ Plants were grown as per *B. napus*, with a total of 15 plants grown for larval screening. Infestation^57^ was carried out at 3 weeks post‐sowing (Fig. 4(A)) when plants possessed four or five true leaves (mean = 4.20). Post‐infestation, a single plant died, leaving 14 plants for larval screening. Two weeks post‐infestation, plants were harvested and larvae were recovered as before.^61^

*Arabidopsis thaliana* larval screening was carried out using the Ws‐0 genotype. Seeds were sown into Levington Advance F2 and stratified at 4 °C for 1 week, before being transferred to the same growth conditions used for *B. napus*. Three weeks post‐stratification, 15 *A. thaliana* plants were re‐potted into 5 cm pots, and left to grow for a further week. Larval infestation was carried out 4 weeks post‐stratification, when rosette‐stage plants possessed 11–18 true leaves (mean = 14.13). Approximately 100 CSFB eggs were set up 2 days before larval infestation. Plants were infested by introducing a single <24‐h‐old larva to the centre of the rosette (Fig. 4(D)), with the introduced larva observed for 10 s to ensure movement. Plants were enclosed in sealed perforated bags (length × width: 30 × 15 cm), ensuring larvae could not move between plants. One week post‐infestation, plants were checked for successful larval infestation, defined as visible larval entry holes and/or mines on the petioles (Fig. 4(E)). Two weeks post‐infestation, plants were harvested and larvae were collected as before.^61^

Recovered larvae (*B. rapa n* = 137; *A. thaliana n* = 9) and <24‐h‐old larvae (*B. rapa n* = 12; *A. thaliana n* = 15) from both experiments were imaged and measured as before.

### 2.6 Statistical analyses

Analyses were conducted using R v.4.5.2 in RStudio v.2026.01.0.^65^, ^66^ Linear models and linear mixed models were fitted using the ‘stats’ and ‘lmerTest’ packages,^67^ respectively. All plots were created with ‘ggplot2’.^68^ Linear model effects were assessed using the *Anova()* function (type II) from the ‘car’ package.^69^ Linear mixed model fixed effects were assessed using the *anova()* function (type II) from the ‘lmerTest’ package,^67^ allowing *P* value calculation via Kenward‐Rogers approximation. Maximal models comprising all explanatory effects were fitted before non‐significant effects (*P* > 0.05) were dropped in a stepwise manner to produce final models (Supporting Information, Table S3). Akaike Information Criteria‐based model selection and assumption checks were performed using the *compare_performance()* and *check_model()* functions from the ‘performance’ package.^70^ Estimated marginal mean (EMM) calculations and Tukey *post hoc* comparisons were performed using the ‘emmeans’ package,^71^ with a significance threshold of *P* < 0.05. All R packages used for analyses are given in Supporting Information, Table S4.

To analyse larval development over time, we fitted live larval recovery, larval size and instar proportion as a function of timepoint. Instar size differences were analysed by fitting larval size as a function of instar. To analyse larval antibiosis across all Brassicaceae genotypes plus those selected for phenotype validation, we fitted larval recovery and larval size as a function of genotype. Development success during the adult emergence experiment was analysed by fitting total adult emergence, time (weeks) to first adult emergence, and mean adult emergence time as a function of genotype. Finally, to analyse larval development in *B. rapa* and *A. thaliana*, we fitted larval size as a function of status (day‐old *versus* recovered). Where necessary, models contained additional terms accounting for experimental design, larval nesting within plants, or confounding factors such as plant weight (Supplementary Methods for full details).

Unless otherwise stated, values in Section 3 represent the raw mean ± standard error.

## 3 RESULTS

### 3.1 Larval development occurs across 4 weeks under laboratory conditions

All infested plants (*n* = 20) showed CSFB larval herbivory symptoms. Live larval recovery was significantly influenced by plant weight (g) at dissection (*F*_(1,15)_ = 5.16, *P* = 0.04) and time point (*F*_(3,15)_ = 17.07, *P* < 0.001), with consistent larval recovery for the first 3 weeks (1 week: 8.00 ± 0.32; 2 weeks: 8.20 ± 0.49; 3 weeks: 7.00 ± 0.63) before decreasing to 3.00 ± 0.95 at 4 weeks post‐infestation (Fig. 1(B)). The same pattern was observed when including dead larvae (Supporting Information, Fig. S7). Larvae grew significantly over time (*F*_(4,12)_ = 134.65, *P* < 0.001), undergoing an ~20.5× size increase from 0.44 ± 0.02 mm^2^ at hatching to 9.05 ± 0.27 mm^2^ at 4 weeks (Fig. 1(C),(E)). Instar composition also changed significantly over time (*F*_(3,16)_ = 368.51, *P* < 0.001; Fig. 1(D)) and larval size significantly increased with instar (*F*_(2,118)_ = 63.19, *P* < 0.001; Supporting Information, Fig. S8). These results suggest that larvae complete development and begin to pupate from 4 weeks post‐infestation under our laboratory conditions.

**Figure 1:**
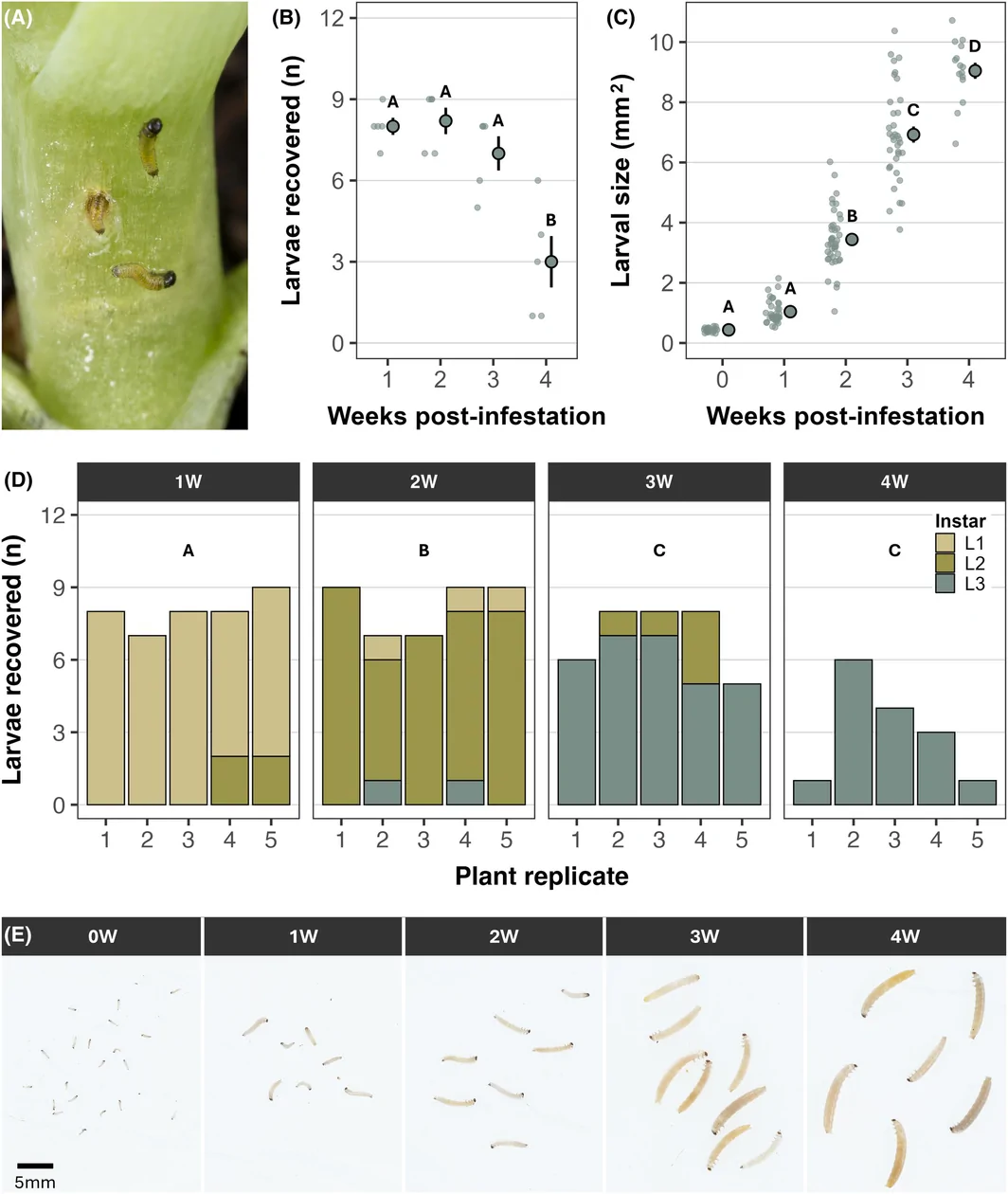
Cabbage stem flea beetle (CSFB; *Psylliodes chrysocephala*) larval development in *Brassica napus* plants under laboratory conditions. (A) Infestation of *B. napus* with <24‐h‐old CSFB larvae. (B) Live larval recovery from infested plants at each time point (1–4 weeks; *n* = 5 plants per time point). (C) Larval size (mm^2^) at each time point. Week 0 represents the size of <24‐h‐old larvae. For plots (B,C), large points with whiskers represent the mean ± standard error, left jittered points represent the raw data, and letters represent significant differences (*P* < 0.05) between time points. (D) Larval instar proportions within each plant across time points. Light green bars are the first instar (L1), mid‐green bars are the second instar (L2) and dark green bars are third instar (L3). Letters within plots represent significant differences in instar composition (*P* < 0.05) between time points. (E) Representative photographs of larvae across time points.

Plant dissections revealed changes in larval location over time, with larvae distributed across the stem, cotyledons and petioles at 1 week post‐infestation, before feeding almost exclusively within petioles from 2 to 4 weeks (Supporting Information, Fig. S9). Petiole sharing was frequently observed, with 42.47% of infested petioles (31 of 71) containing two or more larvae (Supporting Information, Fig. S10).

### 3.2 Potential larval antibiosis in a genetically diverse *B. napus* population

All plants (*n* = 312) showed larval herbivory symptoms. Along with plant weight (g) at evacuation (*F*_(1,144)_ = 6.72, *P* = 0.01), larval survival was significantly influenced by genotype (*F*_(97,194)_ = 1.83, *P* < 0.001; Table 1), with lowest survival in *S. alba* G1 and highest survival in *B. napus* 84 (Fig. 2(A); Supporting Information, S11(A)). Importantly, larval survival was significantly lower (*t*_(1,202)_ = −5.33, *P* < 0.001) in the *S. alba* positive control than in the negative control (Supporting Information, Figs S12(A) and S13), demonstrating expected differences in CSFB larval survival between *S. alba* and *B. napus*.^56^ Excluding *S. alba*, EMM [95% confidence interval (CI)] larval survival ranged from 4.24 (2.18–6.31) to 10.40 (8.34–12.47) across the 97 *B. napus* genotypes (Fig. 2(A)), suggesting that genetic variation for CSFB larval antibiosis may exist in *B. napus*. Using our larval survival distribution, we assigned three genotypes as resistant [EMM (95% CI) larval survival: 67 = 4.24 (2.18–6.31); 61 = 4.40 (2.33–6.47); 33 = 4.62 (2.55–6.68)] and three as susceptible [EMM (95% CI) larval survival: 14 = 9.35 (7.28–11.41); 89 = 9.97 (7.91–12.03); 84 = 10.40 (8.34–12.47)]. Importantly, larval survival was not significantly different between the three resistant genotypes and *S. alba*, but larval survival was significantly lower in the three resistant genotypes compared with the three susceptible genotypes (Supporting Information, Fig. S13).

**Figure 2:**
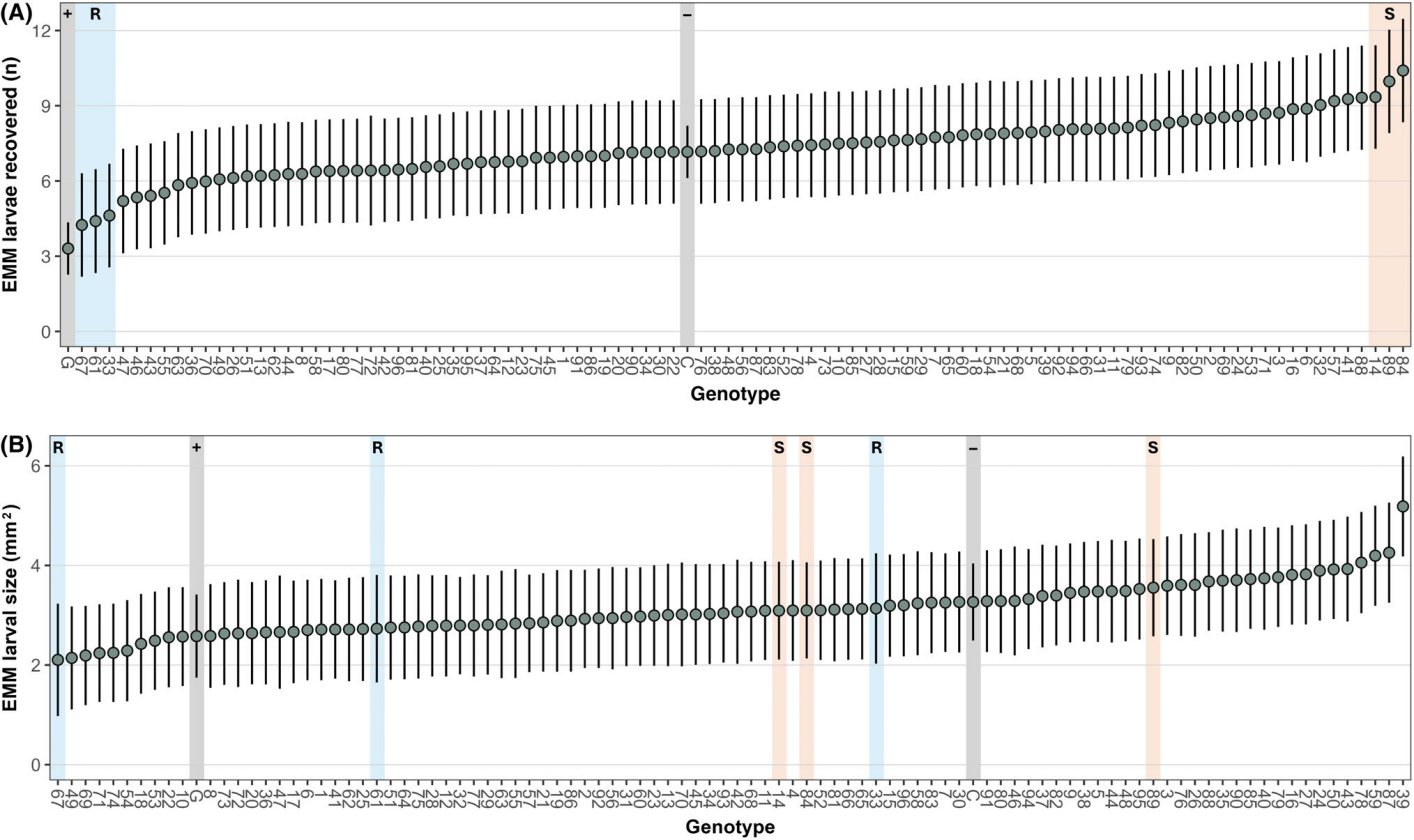
Per‐genotype estimated marginal mean (EMM) ± estimated 95% confidence intervals for cabbage stem flea beetle (*Psylliodes chrysocephala*) larval survival (A) and larval size (mm^2^) (B) across 98 Brassicaceae genotypes (97 *Brassica napus* and 1 *Sinapis alba*). Larval survival is defined as the number of larvae recovered from a plant 2 weeks post‐infestation with 12 larvae. Genotypes are arranged from lowest to highest EMM. Blue and peach highlighted bars represent genotypes identified as resistant (R) and susceptible (S), respectively. Grey highlighted bars represent the positive (G = *S. alba* Elsoms G1) and negative (C = *B. napus* KWS Campus) controls.

**Table 1:** Fixed effect summaries for cabbage stem flea beetle (*Psylliodes chrysocephala*) larval survival and larval size across the 98 Brassicaceae plant genotypes (97 *Brassica napus* and 1 *Sinapis alba*) screened for larval antibiosis

Response	Fixed effect	*F*	Num. df	Den. df	*P* value
Larval survival	**Genotype**	1.83	97	194	**<0.001**
	**Plant weight**	6.72	1	144	**0.01**
	Replicate	1.15	2	6	0.37
Larval size	**Genotype**	1.73	97	170	**<0.001**
	Replicate	2.51	2	8	0.14

Num. df, numerical degrees of freedom; Den. df, Kenward‐Roger estimated denominator degrees of freedom. Significant fixed effects (*P* < 0.05) are given in bold.

Larval size (mm^2^) was not influenced by plant weight or larval density within plants but did vary significantly by genotype (*F*_(97,170)_ = 1.73, *P* < 0.001; Table 1), with smallest larvae in *B. napus* 67 and largest larvae in *B. napus* 39 (Fig. 2B; Supporting Information, S11(B)). There was no significant difference in larval size between control genotypes (Supporting Information, Fig. S12(B)) or between any of the resistant and susceptible genotypes identified by larval survival (Supporting Information, Fig. S14). Using per‐genotype raw means, we found a weak correlation between larval survival and larval size (*F*_(1,96)_ = 4.11, *P* = 0.05; Supporting Information, Fig. S15(A)), suggesting that larvae may develop faster in genotypes in which survival is higher. However, this correlation was not present when using per‐genotype EMMs (*F*_(1,96)_ = 4.11, *P* = 0.09; Supporting Information, Fig. S15(B)). Per‐genotype raw and estimated means for larval survival and size can be found in Supporting Information, Tables S5–S8.

### 3.3 Phenotype validation did not confirm the presence of antibiosis

All plants in the phenotype validation experiment (*n* = 72) showed larval herbivory symptoms. Larval survival was again influenced by both plant weight (g) at evacuation (*F*_(1,58)_ = 6.02, *p* = 0.02) and genotype (*F*_(7,59)_ = 17.77, *P* < 0.001; Table 2), with lowest survival in *S. alba* G1 and highest survival in *B. napus* 84 (Fig. 3(A)). However, no significant differences in larval survival were found across any of the initially identified resistant and susceptible genotypes (Fig. 3(A)), with the plant genotype effect driven entirely by lower larval survival in *S. alba* compared with *B. napus*.

**Table 2:** Fixed effect summaries for cabbage stem flea beetle (*Psylliodes chrysocephala*) larval survival, larval size, total adult emergence, and adult emergence timing across the Brassicaceae genotypes (seven *Brassica napus* and one *Sinapis alba*) selected for larval antibiosis phenotype validation

Response	Fixed effect	*F*	Num. df	Den. df	*P* value
Larval survival	**Genotype**	17.77	7	59	**<0.001**
	**Plant weight**	6.02	1	58	**0.02**
	Block	3.72	2	3	0.13
Larval size	**Genotype**	6.29	7	59	**<0.001**
	Block	2.60	2	2	0.22
Total adults	Genotype	1.08	5	52	0.38
	Block	2.62	1	52	0.11
Weeks to first adult emergence	Genotype	1.26	5	52	0.29
	Block	0.65	1	52	0.42
Mean adult emergence time (weeks)	Genotype	0.73	5	52	0.61
	Block	1.74	1	52	0.19

Num. df, numerical degrees of freedom; Den. df, Denominator degrees of freedom (Kenward‐Roger estimated for larval survival and larval size responses). Significant fixed effects (*P* < 0.05) are given in bold.

**Figure 3:**
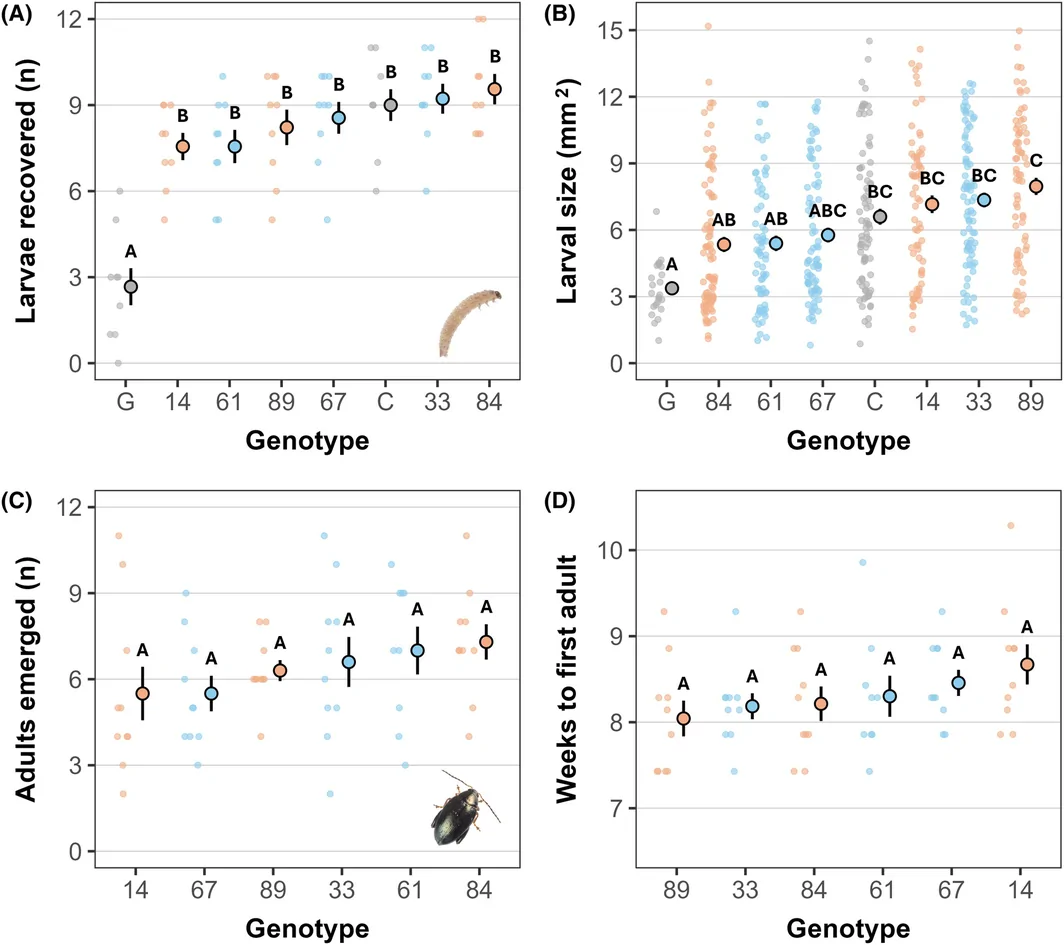
Cabbage stem flea beetle (*Psylliodes chrysocephala*) larval survival (A), larval size (B), and adult emergence (C,D) across eight selected Brassicaceae plant genotypes (seven *Brassica napus* and one *Sinapis alba*). (A) Larval survival, defined as the number of larvae recovered from plants 2 weeks post‐infestation with 12 larvae, across eight Brassicaceae genotypes. (B) Larval size (mm^2^) across eight Brassicaceae genotypes. (C) Total adult emergence across six *B. napus* genotypes. (D) Time (weeks) to first adult beetle emergence across six *B. napus* genotypes. Genotypes are ordered from lowest to highest mean. Blue and peach points represent genotypes from the Brassicaceae population larval antibiosis screen that were initially identified as resistant and susceptible, respectively. Grey points represent the positive (G = *S. alba* Elsoms G1) and negative (C = *B. napus* KWS Campus) controls. Large points with whiskers represent the mean ± standard error, left jittered points represent the raw data, and letters represent significant differences (*P* < 0.05) between genotypes.

There was also a significant effect of genotype on larval size (mm^2^) (*F*_(7,59)_ = 17.77, *P* < 0.001; Table 2), with smallest larvae in *S. alba* G1 and largest larvae in *B. napus* 89 (Fig. 3(B)). Unlike the diversity screen, larvae were significantly smaller (*t*_(1,94)_ = −3.73, *P* < 0.01) in the *S. alba* positive control than the negative control (Fig. 3(B)), corroborating previous observations of slower larval development in *S. alba*.^56^ Interestingly, we also observed significant differences in larval size between *B. napus* genotypes, with genotypes 84 and 61 hosting smaller larvae than genotype 89 (84–89: *t*_(1,51)_ = −3.90, *P* < 0.01; 61–89: *t*_(1,56)_ = −3.81, *P* < 0.01) and no larval size differences between *S. alba* and *B. napus* genotypes 84, 61, and 67 (Fig. 3(B)).

All plants in the adult emergence (*n* = 60) experiment showed larval herbivory symptoms. A mean of 6.36 ± 0.30 adult beetles successfully emerged across all plants (equivalent to a 53% larvae‐to‐adult success rate; Fig. 3(C)), with mean first adult emergence at 8.31 ± 0.08 weeks after infestation (Fig. 3(D)). Consistent with the phenotype validation experiment, there was no significant effect of *B. napus* genotype on total adult emergence (*F*_(5,52)_ = 1.08, *P* = 0.38), weeks to first adult emergence (*F*_(5,52)_ = 1.26, *P* = 0.29; Table 2 and Fig. 3(C),(D)), or mean adult emergence time (*F*_(5,52)_ = 0.73, *P* = 0.61; Supporting Information, Fig. S16). Per‐genotype raw means for all variables measured during both experiments can be found in Tables S9–S13.

### 3.4 Larval screening can be successfully performed in model species

All 15 infested *B. rapa* R‐o‐18 plants showed larval herbivory symptoms. Two weeks post‐infestation, a mean of 9.79 ± 0.30 larvae were recovered from the 14 surviving plants (Fig. 4(B)). Larvae significantly increased in size (*t*_(1,11)_ = 5.19, *P* < 0.001), undergoing a ~10.5× size increase from 0.45 ± 0.01 mm^2^ at hatching to 4.71 ± 0.13 mm^2^ as recovered larvae (Fig. 4(C)).

**Figure 4:**
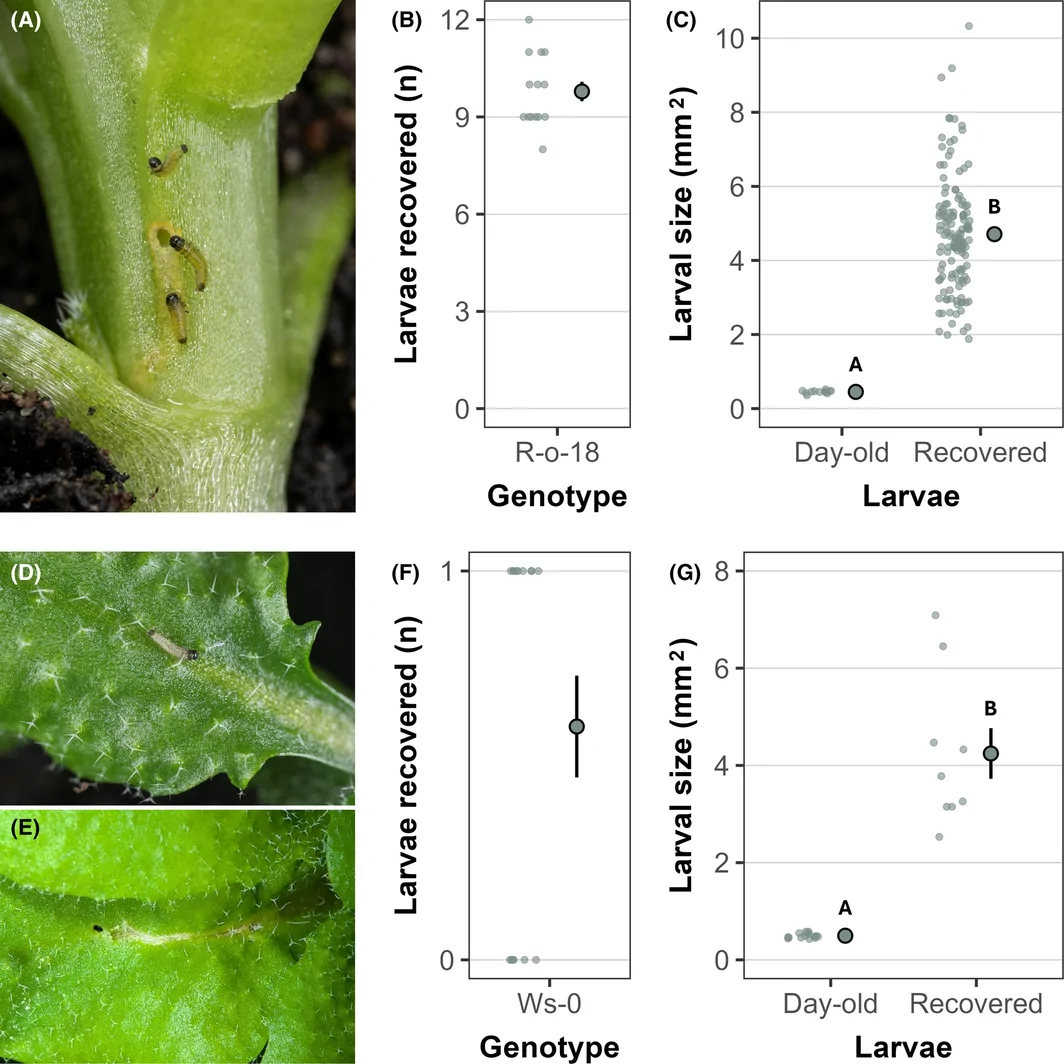
Cabbage stem flea beetle (*Psylliodes chrysocephala*) larval recovery and development within *Brassica rapa* R‐o‐18 (A–C) and *Arabidopsis thaliana* Ws‐0 (D–G) plants. (A) Infestation of *B. rapa* with <24‐h‐old larvae. (B) Larval recovery from *B. rapa* plants (*n* = 14) 2 weeks post‐infestation. (C) Size (mm^2^) of day‐old larvae *versus* larvae recovered from *B. rapa* plants. (D) Infestation of *A. thaliana* with a single <24‐h‐old larva. (E) Larval herbivory damage on the midrib of an *A. thaliana* leaf 1 week post‐infestation. (F) Recovery rate (0/1) of larvae from *A. thaliana* plants (*n* = 15). (G) Size of <24‐h‐old larvae *versus* larvae recovered from *A. thaliana* plants. For plots (B–C, F–G), large points with whiskers represent the mean ± standard error, left jittered points represent the raw data, and letters represent significant differences (*P* ≤ 0.05) based on larval status.

Fourteen of the 15 infested *A. thaliana* plants (93.33%) presented larval herbivory symptoms and, 2 weeks post‐infestation, larvae were recovered from nine plants (60%; Fig. 4(F)). Larvae significantly increased in size (*t*_(1,7)_ = 2.28, *P* = 0.05), developing ~8.5× in size from 0.50 ± 0.01 mm^2^ at hatching to 4.25 ± 0.52 mm^2^ as recovered larvae (Fig. 4(G)). Successful larval screening in *B. rapa* R‐o‐18 and *A. thaliana* Ws‐0 suggests that the genetic resources available for both models can be exploited to study CSFB larval development and resistance genetics.

## 4 DISCUSSION

CSFB larval herbivory imposes severe yield penalties on OSR growth, with larval antibiosis representing a key breeding target. Despite this, the challenging nature of insect resistance phenotyping has limited attempts to explore CSFB resistance.^56^ Our research aimed to address this by producing a laboratory‐based method for controlled larval antibiosis screening,^57^ before: (i) tracking larval development in *B. napus*; (ii) exploring larval antibiosis across a large Brassicaceae population; and (iii) testing larval screening in two model Brassicaceae relatives—*B. rapa* and *A. thaliana*. We showed that, under our growth conditions, larvae complete development from 4 weeks post‐infestation, with larval recovery at 2 weeks post‐infestation providing a faster way of scoring larval antibiosis. Larval screening of the Brassicaceae population revealed limited evidence for resistance in *B. napus* but showed consistent support for larval antibiosis in *S. alba*. Finally, larvae were also able to develop in both *B. rapa* and *A. thaliana*, broadening the genetic resources available to understand CSFB–Brassicaceae interactions.

### 4.1 CSFB larval development

CSFB larvae completed development in *B. napus* from 4 weeks post‐infestation, as evidenced by a sharp decrease in larval recovery, a 20‐fold size increase and progression through three instars. Given that we did not search for pupae, an alternative explanation may be that larval death occurred at 4 weeks. However, because later instars are more robust,^43^, ^72^ and adults were first collected at 7 weeks post‐infestation during the adult emergence experiment, this seems unlikely. Importantly, larval recovery remained high at 2 weeks, with larvae significantly increasing in size. Hence, recovery at this time point provides a faster method to phenotype larval antibiosis across Brassicaceae populations.

Despite controlled larval infestation, larval size was highly variable within plants. Given variable distribution of glucosinolates across tissues,^73^, ^74^ larval development may be influenced by feeding location. Interestingly, we found that larvae avoided apical petioles, where glucosinolates are likely more abundant.^74^ Such patterns have been shown in the generalist *Spodoptera littoralis* ^73^ but whether the same effects exist for specialists such as CSFB is unknown. Larval development may also be affected by larval density, especially as CSFB larvae were found to frequently share petioles. Density‐dependence is a key driver of insect life‐history traits^75^, ^76^ and adverse effects of high density may be stronger in CSFB given that larvae feed internally. Hence, density‐dependent effects on CSFB larval development and life‐history represent an interesting avenue for future research.

### 4.2 Absence of CSFB larval antibiosis across *Brassica napus*

Our initial larval diversity screen identified weak genotype effects on larval survival across the *B. napus* population. However, in follow‐up experiments with larger per‐genotype sample sizes, differences between initially identified ‘resistant’ and ‘susceptible’ genotypes were no longer present, with similar larval survival, larval size, adult emergence and emergence timing found across genotypes. Importantly, the screened *B. napus* population shows variability across other traits.^58^, ^59^, ^60^ Hence, if CSFB larval antibiosis did exist in this panel we should have been able to identify it. This is a crucial finding for the field, because researchers and breeders alike invest substantial resources trying to identify larval antibiosis under field conditions. However, if we cannot find larval antibiosis across a large population with highly controlled phenotyping approaches, it seems unlikely that field trials will provide any promising candidates. A lack of CSFB larval antibiosis in *B. napus* was also shown in Döring and Ulber,^56^ albeit in a much smaller population (4 genotypes *versus* 97 in the current study). Further, *B. napus* appears to lack resistance to multiple insect pests,^23^, ^38^ possibly because of limited genetic diversity after hybridisation followed by selection against glucosinolates during oilseed domestication.^25^, ^32^, ^33^, ^34^, ^35^ All genotypes examined in this study have undergone domestication but, given the inclusion of swedes and kales, not necessarily glucosinolate‐based selection. Hence, it is possible that insect resistance traits besides glucosinolate production have been lost during *B. napus* domestication. However, a thorough comparative investigation between *B. napus* and insect‐resistant relatives or feral *Brassica* species is required to confirm this.

Our study prioritised larval survival over larval size as a measure of larval antibiosis. This was because our phenotyping method involved larval recovery using the Berlese method.^61^ As a result, we were able to screen large numbers of plants at the expense of accurate larval size, given larvae can continue developing within plants before recovery.^61^ We are unable to distinguish between larval death within the plant *versus* larvae failing to penetrate the plant. However, given that all infested plants showed signs of larval herbivory symptoms on the final day, and desiccated larvae were never found on the plant exterior, we are confident that larval survival represents mortality experienced in the plant. Larval antibiosis may take place later than 2 weeks post‐infestation but, given that antibiosis in *S. alba* was consistently observed at 2 weeks, we deem this unlikely.

In the current study, we only assessed the presence of larval antibiosis across *B. napus* genotypes. However, alternative measures of CSFB resistance such as antixenosis and tolerance^18^, ^21^, ^22^ to both adults and larvae across *B. napus* diversity remain untested. For instance, oviposition preference differences across Brassicaceae species have been shown in multiple insects, including cabbage butterflies (*Pieris brassicae* and *Pieris rapae*),^77^ cabbage seedpod weevil (*Ceutorhynchus obstrictus*)^78^ and pollen beetle (*Meligethes aeneus*).^79^ Oviposition preferences for CSFB are currently unknown; however, oviposition rate may depend on the quality of the host plant and the availability of digestible carbohydrates.^80^ Further, tolerance to low larval loads (infestation of fewer than five larvae per plant) has been demonstrated in a single *B. napus* genotype, with yield declines observed with increasing larval density.^43^ However, per‐genotype relationships between larval density and plant yield are unexplored. Hence, although larval antibiosis is unlikely to exist with *B. napus*, alternative mechanisms of plant resistance may yet be discovered.

### 4.3 Brassicaceae relatives can inform CSFB larval resistance

*Sinapis alba*, a relative of *B. napus*, consistently demonstrated CSFB larval antibiosis, with lower larval survival and smaller larvae compared with the screened *B. napus* genotypes. Promisingly, CSFB larval antibiosis has also been reported in a separate *S. alba* genotype,^56^ suggesting that larval resistance may be a shared trait across *S. alba* genetic diversity. Future work should look to confirm whether larval antibiosis is the rule in *S. alba* by applying our screening methodology^57^ to a genetically diverse population. In addition, because we only measured larval survival and size at 2 weeks post‐infestation, it would be interesting to see whether adults can successfully emerge from *S. alba* and adults suffer any adverse effects, such as reduced fecundity or lifespan.^81^ *Sinapis alba* shows resistance to a range of other insect pests,^82^, ^83^, ^84^ and, in some cases, this resistance has been successfully tracked to quantitative trait loci (QTL) and introgressed into OSR.^85^, ^86^ For instance, a mapping population derived from a resistant × susceptible *S. alba* cross allowed the identification of a QTL explaining 10% of heritable variation associated with cabbage seedpod weevil (*C. obstrictus*) resistance.^85^ Hence, identifying the metabolic and genetic factors associated with CSFB resistance in *S. alba* and introgressing these traits into *B. napus* may represent a key strategy in breeding CSFB‐resistant OSR.

In the current study, we showed that CSFB larvae can successfully infest and develop in both *B. rapa* R‐o‐18 and *A. thaliana* Ws‐0. Alongside *S. alba*, both *B. rapa* and *A. thaliana* may also provide insights into CSFB resistance. As the progenitors of *B. napus*, genetic diversity is far higher in both *B. rapa* and *B. oleracea*,^25^ and both species may contain sources of CSFB resistance. An interesting approach could be to screen populations of both species for CSFB resistance before performing crosses to pyramid genes and produce re‐synthesised, resistant *B. napus* genotypes. Such approaches have previously been applied to re‐synthesise *B. napus* with resistance to the fungal pathogen *Verticillium longisporum*,^87^, ^88^ while re‐synthesised *B. napus* has also shown increased resistance to rape stem weevil (*C. napi*).^89^ In addition, *B. rapa* R‐o‐18 offers a selection of TILLING mutants,^64^ providing a rich resource for testing gene function in relation to larval resistance.

CSFB larval screening in *Arabidopsis* also offers a promising alternative to identify resistance genes. Alongside the huge genetic diversity available in *Arabidopsis*, larval screening could have a far higher throughput than in *Brassica* species, given the requirement for fewer larvae (1 larva per plant in *Arabidopsis versus* 12 larvae in *Brassica*), fewer infestation days (1 day in *Arabidopsis versus* 4 days in *Brassica*) and less space. Plant–insect association studies across diverse *Arabidopsis* populations have identified candidate genes for cabbage butterfly (*P. brassicae*) oviposition,^90^ cabbage whitefly (*Aleyrodes proletella*) survival and fecundity,^91^ and diamondback moth (*Plutella xylostella*) larval herbivory.^92^ Such approaches could therefore be used to identify CSFB resistance candidates in *Arabidopsis* before searching for homologues in *Brassica* genomes. Further, the rich diversity of knock‐out and over‐expression lines in *Arabidopsis* offers a convenient method for testing CSFB resistance gene function.

## 5 CONCLUSIONS

We outline a method for highly controlled CSFB larval screening in Brassicaceae and, by applying this method to a diverse collection of Brassicaceae genotypes, show limited evidence for larval antibiosis in *B. napus* but consistent antibiosis in *S. alba*. These findings highlight the need for researchers and breeders to look beyond *B. napus* genetic diversity to uncover genes involved in CSFB resistance, with Brassicaceae relatives representing a promising resource. In addition, the suitability of *B. rapa* and *A. thaliana* for larval screening provides further genetic tools to mine for CSFB resistance. Finally, beyond the use of these methods for gene and trait discovery in OSR breeding, we also suggest that the CSFB–Brassicaceae system could provide fundamental insights into plant–insect interactions for insects with larval mining strategies.

## AUTHOR CONTRIBUTIONS

REB: Conceptualization (supporting); Methodology (lead); Investigation (lead); Data curation (lead); Formal analysis (lead); Visualization (lead); Funding acquisition (supporting); Supervision (supporting); Writing – original draft (lead); Writing – review & editing (equal). CC: Methodology (supporting); Investigation (supporting); Writing – review & editing (supporting). SP: Conceptualization (supporting); Funding acquisition (supporting); Supervision (supporting); Writing – review & editing (supporting). RW: Conceptualization (lead); Funding acquisition (lead); Supervision (lead); Writing – review & editing (equal).

## Supporting information


**Data S1.** Supporting Information.

**Figure S1.** Layout for the cabbage stem flea beetle (*Psylliodes chrysocephala*; CSFB) larval development time course experiment performed in a single *Brassica napus* genotype (Apex‐93_5 × Ginyou_3 DH). Twenty plants infested with 12 CSFB larvae each were grown in a 4 × 5 row‐column matrix, with each column containing a single replicate of each dissection timepoint: 1 W = plant dissected 1 week after larval infestation; 2 W = plant dissected 2 weeks after larval infestation; 3 W = plant dissected 3 weeks after larval infestation; 4 W = plant dissected 4 weeks after larval infestation.
**Figure S2.** Setup for imaging and measuring cabbage stem flea beetle (*Psylliodes chrysocephala*; CSFB) larvae recovered from Brassicaceae plants (*Brassica napus*, *B. rapa*, *Sinapis alba*, and *Arabidopsis thaliana*). (A) Photography setup comprising an overhead camera mount (Kaiser RS1, Kaiser Fototechnik, Buchen, Germany), DSLR camera (Nikon Z6 with AF Micro Nikkor 60 mm lens, Nikon, Tokyo, Japan), A3 LED light pad (AGM, Manchester, UK), two overhead LED lights (Raleno PLV‐S116, KEJI, Shenzen City, China), and a scale/colour chart (Wex Photo Video, Norwich, UK). (B) Raw image of CSFB larvae recovered from an infested *B. rapa* R‐o‐18 plant, including label (genotype + replicate number) and scale. (C) Cropped raw image, containing only the larvae to be measured. (D) Binary conversion of larval image, allowing measurement of larval size (in mm^2^) using the ‘Analyse Particles’ function in ImageJ v1.52a. Scale bar represents 5 mm for (C,D).
**Figure S3.** Block layouts for phenotyping 96 *Brassica napus* genotypes for cabbage stem flea beetle (*Psylliodes chrysocephala*) larval antibiosis. *B. napus* genotypes were randomised across a temporal 12‐block alpha design (plots per block (*k*) = 24, replicates per genotype (*r*) = 3, blocks per replicate (*s*) = 4), and grown across two CER shelves, with a new block beginning every 1–9 weeks. Every block also contained a negative *B. napus* control (KWS Campus = C) and a positive *Sinapis alba* control (Elsoms G1 = G). Each block was split across two cages, with each cage setup in a 5 × 3 row‐column matrix. For block 8, two genotypes (57 and 72) died prior to larval infestation (cells with red strikethrough text). These genotypes were therefore placed into later blocks (57 into block 11 and 72 into block 12; cells with blue text). Each number (1–96) represents a *B. napus* genotype from the RIPR diversity set (Table S1).
**Figure S4.** Block layouts for screening eight Brassicaceae genotypes (seven *Brassica napus* and a single *Sinapis alba*; Table S1) for cabbage stem flea beetle (*Psylliodes chrysocephala*) larval antibiosis phenotype validation. Genotypes included the ‘resistant’ *B. napus* (genotypes 33, 61 and 67), the ‘susceptible’ *B. napus* (genotypes 14, 84 and 89), the negative *B. napus* control (KWS Campus = C), and the positive *S. alba* control (Elsoms G1 = G). Genotypes were randomised across three blocks, with a new block starting every 1–3 weeks. All blocks were grown across two CER shelves, with each block containing three replicates of each genotype. Each block was split across two cages, with each cage set up in a 4 × 3 row‐column matrix.
**Figure S5.** Comparison of cabbage stem flea beetle (*Psylliodes chrysocephala*) larval survival in the two control genotypes (G = *Sinapis alba* Elsoms G1; C = *Brassica napus* KWS Campus) between the Brassicaceae genotype screening experiment (GS, left) and phenotype validation experiment (PV, right). Larval survival is defined as the number of CSFB larvae recovered from a plant 2 weeks after infestation with 12 larvae. Large points with whiskers represent the mean ± standard error and jittered points to the left represent the raw data. Statistical analyses were not run to compare differences in larval survival between the two experiments given that the independent experiments were run with different designs (Figs S3 and S4).
**Figure S6.** Block layouts for screening six *Brassica napus* genotypes for cabbage stem flea beetle (*Psylliodes chrysocephala*) larval antibiosis (*via* adult beetle emergence). Genotypes included the ‘resistant’ *B. napus* (genotypes 33, 61 and 67), and the susceptible *B. napus* (genotypes 14, 84, and 89). Genotypes were randomised across two blocks, with a new block starting each week. Both blocks were grown across two CER shelves, with each block containing five replicates of each genotype. Each block was split across two cages, with each cage set up in a 5 × 3 row‐column matrix. Full genotype details can be found in Table S1.
**Figure S7.** Total recovery of cabbage stem flea beetle (*Psylliodes chrysocephala*) larvae from *Brassica napus* (Apex‐93_5 × Ginyou_3 DH) plants infested with 12 larvae each. Plants were dissected over four timepoints (1–4 weeks) following infestation, with five plants dissected per timepoint. Grey bars = total live larvae found within each plant; black bars = total dead larvae found within each plant.
**Figure S8.** Size of cabbage stem flea beetle (*Psylliodes chrysocephala*; CSFB) larvae recovered from infested *Brassica napus* (Apex‐93_5 × Ginyou_3 DH) plants by larval instar. Large points with whiskers represent the mean ± standard error, jittered points to the left represent the raw data, and letters represent significant differences (*P* < 0.05) between larval instars.
**Figure S9.** Location of cabbage stem flea beetle (*Psylliodes chrysocephala*) larvae recovered from *Brassica napus* (Apex‐93_5 × Ginyou_3 DH) plants infested with 12 larvae each. Plants were dissected over four timepoints (1–4 weeks) following infestation, with five plants dissected per timepoint. S = stem; C1‐2 = cotyledons; P1‐12 = true leaf petioles, with P1 representing the first true leaf petiole and sequential numbers representing the next true leaf (i.e., high P numbers represent the youngest true leaf petioles, closest to the plant apex). Large points with whiskers represent the mean ± standard error.
**Figure S10.** Number of cabbage stem flea beetle (*Psylliodes chrysocephala*) larvae per petiole in *Brassica napus* (Apex‐93_5 × Ginyou_3 DH) plants infested with 12 larvae each. Plants were dissected over four timepoints (1–4 weeks) following infestation, with five plants dissected per timepoint. Hence, bars within each timepoint represent the total number of dissected petioles across all plants within a timepoint. Occurrences of two or more larvae per petiole are defined as petiole sharing.
**Figure S11.** Per genotype mean ± standard error for cabbage stem flea beetle (*Psylliodes chrysocephala*) larval survival (A) and larval size (B) across 98 Brassicaceae genotypes (97 *Brassica napus* and a single *Sinapis alba*; Table S1). Larval survival is defined as the number of CSFB larvae recovered from a plant 2 weeks after infestation with 12 larvae. For both plots, genotypes are arranged from lowest to highest mean. The dashed horizontal line with grey shading represents the mean ± standard error for all data. Blue and peach highlighted bars represent genotypes identified as resistant (R) and susceptible (S), respectively. Grey highlighted bars represent the positive (G = *S. alba* Elsoms G1) and negative (C = *B. napus* KWS Campus) controls, respectively.
**Figure S12.** Cabbage stem flea beetle (*Psylliodes chrysocephala*) larval survival (A) and larval size (B) in the two control genotypes (G = *Sinapis alba* Elsoms G1; C = *Brassica napus* KWS Campus) used for the *B. napus* population larval antibiosis screen. Larval survival is defined as the number of CSFB larvae recovered from a plant 2 weeks after infestation with 12 larvae. Large points with whiskers represent the mean ± standard error, jittered points to the left represent the raw data, and letters represent significant differences (*P* < 0.05) between genotypes.
**Figure S13.** Per genotype estimated marginal means and contrasts for cabbage stem flea beetle (*Psylliodes chrysocephala*; CSFB) larval survival across eight selected Brassicaceae genotypes (seven *Brassica napus* and a single *Sinapis alba*; Table S1) from the Brassicaceae population larval antibiosis screen. Larval survival is defined as the number of CSFB larvae recovered from a plant 2 weeks after infestation with 12 larvae. (A) Per genotype EMM ± estimated 95% confidence intervals for larval survival across resistant (blue), susceptible (peach), and control (grey) plant genotypes (G = *S. alba* Elsoms G1; C = *B. napus* KWS Campus). Letters represent significant differences (*P* < 0.05) between genotypes. (B) Contrast matrix for larval survival across the eight genotypes. Values in the black diagonal boxes represent EMMs for each genotype, values in the left‐hand triangle represent EMM contrasts between genotypes (darker grey = larger contrast), and values in the right‐hand triangle represent significance thresholds between genotype comparisons (darker green = smaller *P* value).
**Figure S14.** Per genotype estimated marginal means and contrasts for cabbage stem flea beetle (*Psylliodes chrysocephala*; CSFB) larval size across eight selected Brassicaceae genotypes (seven *Brassica napus* and a single *Sinapis alba*; Table S1) from the Brassicaceae population larval antibiosis screen. (A) Per genotype EMM ± estimated 95% confidence intervals for larval size across resistant (blue), susceptible (peach), and control (grey) plant genotypes (G = *S. alba* Elsoms G1; C = *B. napus* KWS Campus). Letters represent non‐significant differences (*P* > 0.05) between genotypes. (B) Contrast matrix for larval size across the eight genotypes. Values in the black diagonal boxes represent EMMs for each genotype, values in the left‐hand triangle represent EMM contrasts between genotypes (darker grey = larger contrast), and values in the right‐hand triangle represent significance thresholds between genotype comparisons (darker green = smaller *P* value).
**Figure S15.** Per genotype relationships between cabbage stem flea beetle (*Psylliodes chrysocephala*) larval survival and larval size for all 98 Brassicaceae genotypes (97 *Brassica napus* and a single *Sinapis alba*; Table S1) from the Brassicaceae population larval antibiosis screen. (A) Raw mean larval size by raw mean larval survival across all 98 Brassicaceae genotypes. (B) Estimated marginal mean (EMM) larval size by EMM larval survival across all 98 Brassicaceae genotypes. For both plots, each point represents one of the 98 genotypes included in the diversity screen, with resistant (blue points), susceptible (peach points), and control (grey points) genotypes individually labelled (G = *S. alba* Elsoms G1; C = *B. napus* KWS Campus).
**Figure S16.** Cabbage stem flea beetle (*Psylliodes chrysocephala*) mean adult emergence time (in weeks) across the six selected *Brassica napus* genotypes used for the adult emergence experiment. Genotypes are ordered from lowest to highest mean. Blue and peach points represent genotypes from the *B. napus* population screen that were initially identified as resistant and susceptible, respectively (Fig. 2). Large points with whiskers represent the mean standard error, jittered points to the left represent the raw data, and letters represent non‐significant differences (*P* > 0.05) between groups.
**Table S1.** Genotype information for the 98 Brassicaceae genotypes (97 *Brassica napus* plus a single *Sinapis alba*) screened for cabbage stem flea beetle (*Psylliodes chrysocephala*) larval antibiosis. Blue and peach highlighted rows indicate genotypes initially identified as resistant and susceptible, respectively. Grey highlighted rows indicate the two control genotypes. BnASSYST ID represents the corresponding genotype ID from the Renewable Industrial Products from Rapeseed (RIPR) panel.
**Table S2.** Alpha experimental design for screening 96 *Brassica napus* genotypes for cabbage stem flea beetle (*Psylliodes chrysocephala*) larval antibiosis. Alpha parameters: plots per block (*k*) = 24, replicates per genotype (*r*) = 3, blocks per replicate (*s*) = 4. Each block also contained a negative *B. napus* control (C) and a positive *Sinapis alba* control (G). In block 8, two genotypes died prior to larval infestation (cells with red strikethrough text) and so were regrown and placed into later blocks (cells with blue text). Genotype information for all 98 Brassicaceae genotypes screened can be found in Table S1.
**Table S3.** Model descriptions, formulas, types, error structures, fits, and sample sizes for the statistical models fitted for cabbage stem flea beetle (*Psylliodes chrysocephala*) larval screening analyses. Models follow the formula: response variable~explanatory variables. For each model, bold black text represents the final fitted model and grey text represents effects included in the maximal model that were dropped due to non‐significance (in the case of fixed effects) or singular fit (in the case of random effects). Statistically significant (*P* < 0.05) terms within models are denoted with an asterisk. See Supplementary Methods for justification of all effects included and retained within fitted models. Type: LM = linear model; LMM = linear mixed model. *R* ^2^ represents the adjusted *R* ^2^ for linear models and the marginal *R* ^2^ for linear mixed models. Parentheses after sample sizes represent sample size nesting, whereby larvae were nested within samples. Note that nesting levels for larval size in the larval diversity screen and phenotype validation models do not match the total number of plants screened due to no larvae being recovered in two instances.
**Table S4.** R packages and versions used for cabbage stem flea beetle (*Psylliodes chrysocephala*) larval screening analyses. All analyses were carried out using R v4.5.2 within Rstudio v2026.01.0. Packages are listed in alphabetical order.
**Table S5.** Raw summary statistics for cabbage stem flea beetle (*Psylliodes chrysocephala*) larval recovery across the 98 Brassicaceae genotypes screened for larval antibiosis. Blue and peach highlighted rows indicate genotypes initially identified as resistant and susceptible, respectively. Grey highlighted rows indicate the two control genotypes. Genotype information for all 98 Brassicaceae genotypes can be found in Table S1.
**Table S6.** Estimated marginal means, standard errors, degrees of freedom, and 95% confidence interval limits for cabbage stem flea beetle (*Psylliodes chrysocephala*) larval recovery across the 98 Brassicaceae genotypes screened for larval antibiosis. Model used for estimation can be found in Table S3. Blue and peach highlighted rows indicate genotypes initially identified as resistant and susceptible, respectively. Grey highlighted rows indicate the two control genotypes. Genotype information for all 98 Brassicaceae genotypes can be found in Table S1.
**Table S7.** Raw summary statistics for cabbage stem flea beetle (*Psylliodes chrysocephala*) larval size across the 98 Brassicaceae genotypes screened for larval antibiosis. Blue and peach highlighted rows indicate genotypes initially identified as resistant and susceptible, respectively. Grey highlighted rows indicate the two control genotypes. Genotype information for all 98 Brassicaceae genotypes can be found in Table S1.
**Table S8.** Estimated marginal means, standard errors, degrees of freedom, and 95% confidence interval limits for cabbage stem flea beetle (*Psylliodes chrysocephala*) larval size across the 98 Brassicaceae genotypes screened for larval antibiosis. Model used for estimation can be found in Table S3. Blue and peach highlighted rows indicate genotypes initially identified as resistant and susceptible, respectively. Grey highlighted rows indicate the two control genotypes. Genotype information for all 98 Brassicaceae genotypes can be found in Table S1.
**Table S9.** Raw summary statistics for cabbage stem flea beetle (*Psylliodes chrysocephala*) larval recovery across the 8 Brassicaceae genotypes selected for the phenotype validation experiment. Blue and peach highlighted rows indicate genotypes initially identified as resistant and susceptible, respectively. Grey highlighted rows indicate the two control genotypes. Genotype information for all Brassicaceae genotypes can be found in Table S1.
**Table S10.** Raw summary statistics for cabbage stem flea beetle (*Psylliodes chrysocephala*) larval size across the 8 Brassicaceae genotypes selected for the phenotype validation experiment. Blue and peach highlighted rows indicate genotypes initially identified as resistant and susceptible, respectively. Grey highlighted rows indicate the two control genotypes. Genotype information for all Brassicaceae genotypes can be found in Table S1.
**Table S11.** Raw summary statistics for cabbage stem flea beetle (*Psylliodes chrysocephala*) adult emergence across the six *Brassica napus* genotypes selected for the adult emergence experiment. Blue and peach highlighted rows indicate genotypes initially identified as resistant and susceptible, respectively. Genotype information for all *B. napus* genotypes can be found in Table S1.
**Table S12.** Raw summary statistics for cabbage stem flea beetle (*Psylliodes chrysocephala*) first adult emergence timing across the six *Brassica napus* genotypes selected for the adult emergence experiment. Blue and peach highlighted rows indicate genotypes initially identified as resistant and susceptible, respectively. Genotype information for all *B. napus* genotypes can be found in Table S1.
**Table S13.** Raw summary statistics for cabbage stem flea beetle (*Psylliodes chrysocephala*) adult mean development time in weeks across the six *Brassica napus* genotypes selected for the adult emergence experiment. Blue and peach highlighted rows indicate genotypes initially identified as resistant and susceptible, respectively. Genotype information for all *B. napus* genotypes can be found in Table S1.

## Data Availability

All data and code required for visualisation and analyses are available from Zenodo (https://doi.org/10.5281/zenodo.18009384).^93^
